# Gastrojejunal Enteral Tube Serving as a Small Bowel Bezoar Nidus

**DOI:** 10.7759/cureus.15266

**Published:** 2021-05-27

**Authors:** Derek G Armstrong, Isha Kaul, Jose A Hernandez, Bruno P Chumpitazi

**Affiliations:** 1 Department of Pediatrics, Baylor College of Medicine, Houston, USA; 2 Department of Pediatrics, Section of Gastroenterology, Hepatology, and Nutrition, Baylor College of Medicine, Houston, USA; 3 Division of Pediatric Gastroenterology, Hepatology, and Nutrition, Texas Children’s Hospital, Houston, USA; 4 Department of Radiology, Baylor College of Medicine, Houston, USA; 5 Division of Interventional Radiology, Texas Children’s Hospital, Houston, USA; 6 Department of Agriculture Research Service, Children’s Nutrition Research Center, Houston, USA

**Keywords:** bezoar, trichobezoar, pica, gastrojejunal enteral tube, trichotillomania, trichophagia, gastroenterology and endoscopy, interventional radiology

## Abstract

Gastrojejunal (GJ) tube placement is indicated in the management of gastric feeding-related intolerance. Though uncommon, GJ complications may occur. We present the case of a five-year-old male with congenital heart disease in which image-guided replacement of a GJ tube was unable to be completed due to a mass adhered to the tip of the tube. The subsequent endoscopic evaluation identified the mass as a hair-based bezoar and the tube was successfully removed. The child was subsequently diagnosed with trichotillomania, trichophagia, and pica. This case illustrates the importance of recognizing bezoar formation as a potential complication of GJ enteric tubes, particularly in children with trichophagia and pica.

## Introduction

Gastrojejunal (GJ) tube placement is a common practice to provide enteral nutrition in patients with gastric feeding intolerance secondary to disorders such as severe gastroesophageal reflux disease (GERD), intractable vomiting, or gastroparesis [1,2]. Children who may benefit from GJ tube feeding may also include those at higher risk for aspiration due to underlying concomitant medical diagnoses related to neurological disorders (e.g., cerebral palsy), congenital heart disease, and chronic lung disease with a history of prematurity [1]. Given the need for the placement of a soft feeding catheter into the small bowel, placement and replacement of GJ tubes are completed via radiology-based image-guided techniques. Unfortunately, GJ tube complications may occur. Known complications of GJ tubes include leaking, obstruction, breakage, or dislodgement. More severe complications such as intussusception or intestinal perforation have also been previously reported [2,3]. In this case report, we present the case of a five-year-old with congenital heart disease, trichotillomania, trichophagia, pica, and GJ-based enteral feeds who presented with a bezoar at the enteric tube tip requiring endoscopic removal.

## Case presentation

**Figure 1:**
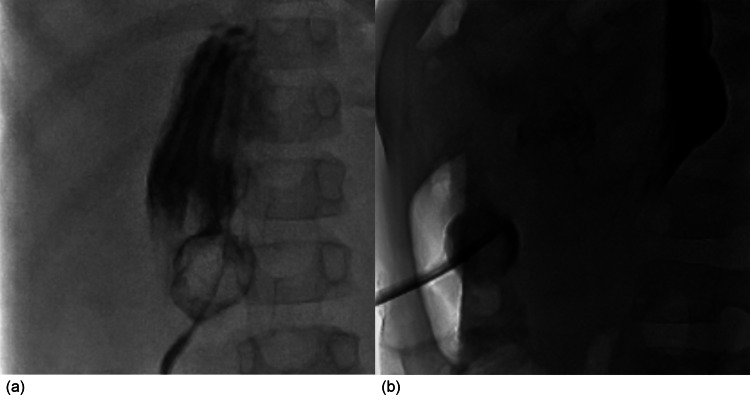
Anteroposterior (a) and lateral (b) fluoroscopic views during GJ exchange showed a rounded structure adhered to the tip of the GJ tube. GJ: gastrojejunal

A five-year-old male with congenital heart disease, developmental delay, and GJ tube dependence presented to his pediatrician’s office for a routine checkup. The patient had been attempting to transition to oral feeding, but it was determined that he still required enteral supplementation with GJ feeds. Although the family did not report problems with the GJ tube, it was noted that he had not had an exchange for eight months, for which he was referred to the interventional radiology (IR) clinic for GJ replacement. The following day he presented to the IR clinic for his GJ exchange. As the interventional radiologist began retracting the GJ tube, he met increasing resistance as the tip of the tube approached the gastrostomy stoma. The GJ tube could not be fully extracted and further attempts to remove the tube led the patient to become increasingly uncomfortable. Additional fluoroscopic evaluation identified a mass surrounding the end of the tube that was preventing successful removal (Figure 1). General surgery and gastroenterology were consulted. The more proximal end of the GJ tube was cut and clamped at the stoma until further endoscopic intervention could be undertaken to identify the mass and attempt extraction.

**Figure 2:**
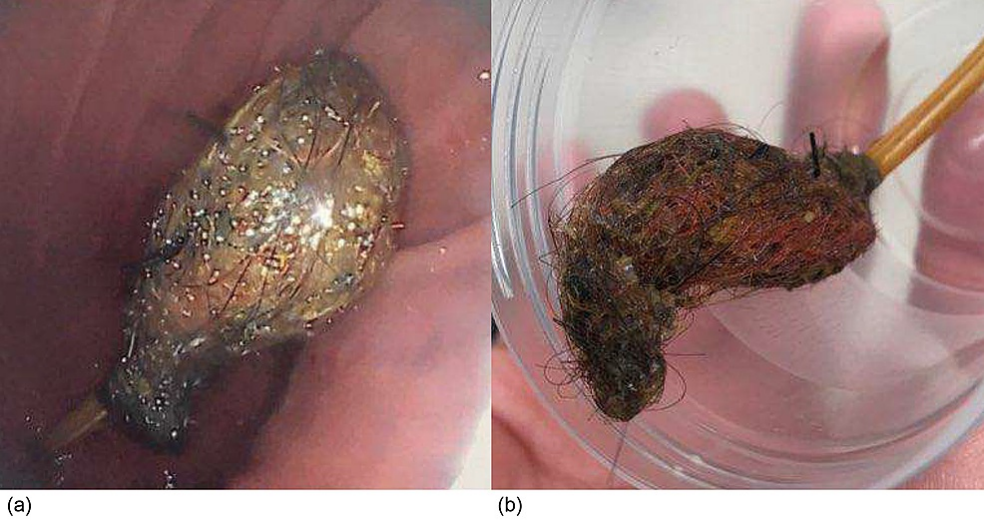
Trichobezoar identified at the end of the GJ tube during upper endoscopy (a) and after removal within a specimen cup (b). GJ: gastrojejunal

During the esophagogastroduodenoscopy, it was found that the tip of the tube was surrounded by a mass of food debris and plastic that was caught in a web of the patient’s hair (Figure 2). The endoscopic team then used an endoscopic grasping net to grasp the bezoar, pushed the remaining GJ tube into the stomach through the stoma, and extracted the remaining portions of the GJ tube and bezoar with the endoscope. A new GJ tube was then successfully placed through the original stoma.

After describing the findings to the family, they subsequently endorsed the patient had been pulling out and eating his own hair and chewing on plastic materials for several years. He was then discharged home with outpatient follow-up with his pediatrician and psychiatry to address his trichotillomania (hair pulling), trichophagia (hair swallowing), and pica (eating non-nutritive substances).

## Discussion

Here, we described the case of a five-year-old boy with congenital heart disease with a GJ tube who developed a bezoar complication identified at the time of attempted GJ replacement secondary to long-term use of a single GJ tube and underlying trichotillomania, trichophagia, and pica. Commonly reported reasons for GJ tube replacement include tube dislodgement, obstruction from formula or medications, or broken or leaking tube components. These complications become more likely the longer a GJ tube is left in place, leading some centers and GJ manufacturers to recommend routine exchange every three to four months [1,2]. More severe GJ complications such as intussusception or intestinal perforation have also been previously reported; however, routine GJ exchange is unlikely to prevent these more serious complications [2,3]. Given the patient described in our case had undergone previous GJ tube exchanges which were successful, we speculate that the patient was at a higher risk for developing a bezoar at the GJ tube due to the prolonged period (eight months) without an exchange. It is possible that a GJ replacement sooner than eight months may have prevented the bezoar from becoming large enough to prevent routine image-guided removal.

Trichotillomania is the recurrent pulling of one’s hair and is often diagnosed in young adolescent females with the age of onset typically 10 to 13 years [4,5]. However, it may occur earlier in patients with developmental delay. As in our case, about 20% of patients with trichotillomania also engage in trichophagia or the compulsive eating of one’s own hair [4,5]. Pica is the craving for and ingestion of non-nutritive, non-food substances that is discordant with cultural practices [6]. It is most commonly known for its association with iron deficiency anemia, but is also commonly seen in patients with developmental delay or intellectual disability as they may have persistent oral exploratory behaviors and be unable to differentiate between edible and inedible objects [6]. The most commonly reported types of pica are geophagia (eating of dirt or clay) and amylophagia (eating of raw starch), and these have been associated with iron deficiency anemia [6]. Individuals with intellectual disability may ingest a wide variety of inedible objects, and in this case the patient was ingesting plastic.

In the setting of the patient’s underlying developmental delay, trichophagia, and pica, his GJ tube served as a nidus for bezoar formation. A bezoar is a concretion of indigestible materials that forms in the gastrointestinal tract [7]. In this case, it was a mix between a trichobezoar consisting of hair and other materials including plastic. Bezoars can be treated chemically, removed endoscopically, or removed surgically if severe and causing intestinal obstruction [7-10]. Lactobezoars (milk-based) have been described in infants which often respond to gastric lavage [11].

Following trichobezoar removal, patients should be referred to psychiatry to prevent recurrence and address the underlying trichotillomania, trichophagia, and pica [4-8]. It has been shown that behavioral therapy, increased parental supervision, and efforts to decrease boredom significantly reduce the severity of pica in patients with intellectual disability [6]. The main treatment for trichotillomania and trichophagia includes habit reversal therapy, but this may be challenging in those with intellectual disability. Pharmacotherapy with N-acetyl cysteine has also been used to reduce the urge for trichotillomania and trichophagia [4,5].

This patient with developmental delay was at an increased risk for having pica. Inquiring further about what the patient was ingesting orally prior to the GJ complication may have alerted his healthcare team to the increased risk for bezoar formation and/or consideration of other enteral feeding methods. In addition, future anticipatory guidance for families of children with GJ tube placement and developmental delay may include discussing the need to identify, and if needed, prevent swallowing of non-nutritive substances.

## Conclusions

This case illustrates the importance of recognizing bezoar formation as a potential complication of GJ enteric tubes, particularly in children with trichophagia and pica. Prolonged periods between GJ exchange may increase the risk of GJ complications such as bezoar formation. Prevention of this complication may include anticipatory guidance to identify and prevent swallowing of non-nutritive substances.
